# Metacognition in functional cognitive disorder: contradictory or convergent experimental results?

**DOI:** 10.1093/braincomms/fcac138

**Published:** 2022-05-27

**Authors:** AJ Larner

**Affiliations:** Department of Brain Repair & Rehabilitation, Institute of Neurology, University College London, London WC1B 5EH, UK

It has previously been hypothesized that functional cognitive disorders (FCDs), at least in some instances, may be a consequence of disordered metacognitive processes.^1,2^ Hence the recent article by Bhome *et al*.^3^ in Brain Communications, which presented evidence supporting this hypothesis, was of great interest. However, Pennington *et al*.^4^ had previously reported that they ‘did not find metacognitive deficits in groups of well characterized patients with FCD’, a study finding not discussed by Bhome *et al*. This former report may thus potentially jeopardise proposed Bayesian and metacognitive models of FCD.^2,5^ How are these apparently contradictory findings to be explained, or possibly reconciled?

Both groups used forced choice (working) memory and (visual) perceptual tasks with trial-by-trial confidence ratings to assess metacognitive efficiency^3^ or efficacy^4^ with a hierarchical meta-d’/d’ model (ideal = 1). Although there were some methodological differences (e.g. exposure time for memory trials), the overall experimental approach was very similar. FCD patients were recruited from different settings (tertiary neuropsychiatry clinical services^3^ versus tertiary referral cognitive disorders clinic^4^), and control groups were constituted from either historical data from healthy controls^3^ or contemporaneously investigated patients with neurodegenerative mild cognitive impairment and healthy individuals.^4^ FCD patients were younger in the neuropsychiatry cohort (mean age 49.2 years) than in the cognitive disorders cohort (57.2 years). As the numbers of FCD participants were small (18 and 20, respectively), it remains a possibility that one study result may reflect a Type I error (detecting an effect that does not exist) and/or that the other study may reflect a Type II error (failing to detect an effect that does exist).

In fact, the experimental findings of the two studies converge rather than contradict. Both studies found that both memory and perceptual task meta-d’/d’ did not differ significantly between groups, with metacognitive efficiency greater for memory than perceptual tasks. Bhome *et al*.^3^ characterize these findings as preserved local (bottom-up) metacognitive efficiency, in contrast with impaired overall or global metacognitive (top-down) efficiency (measures of the latter are not presented in the Pennington paper^4^).

The disconnect or mismatch between global (impaired) and local (intact) metacognition is interpreted in the Bayesian model as abnormal ‘priors’, as per other Bayesian models of functional disorders.^6^ What leads to this proposed pathological decoupling remains to be clarified. One recent testable hypothesis suggests that FCD reflects an ‘overfitting’ of neural networks as a consequence of impaired sleep and dreaming in these patients, which might account for the impairment in global metacognition causing mismatch between memory expectations and memory performance.^7^

## Data availability

Data sharing is not applicable to this article as no new data were created or analysed.

## Competing interests

The authors report no competing interests.

## References

[1] Larner AJ . Dementia screening: a different proposal. Future Neurol. 2018;13:177–179.

[2] Bhome R , McWilliams A, Huntley JD, Fleming SM, Howard RJ. Metacognition in functional cognitive disorder – a potential mechanism and treatment target. Cogn Neuropsychiatry. 2019;24:311–321. 31389291 10.1080/13546805.2019.1651708

[3] Bhome R , McWilliams A, Price G, et al Metacognition in functional cognitive disorder. Brain Commun. 2022;4:fcac041. 35243345 10.1093/braincomms/fcac041 PMC8889108

[4] Pennington C , Ball H, Swirski M, Newson M, Coulthard E. Metacognitive performance on memory and visuospatial tasks in functional cognitive disorder. Brain Sci. 2021;11:1368. 34679432 10.3390/brainsci11101368 PMC8533868

[5] Larner AJ . Functional cognitive disorders (FCD): how is metacognition involved? Brain Sci. 2021;11:1082. 34439701 10.3390/brainsci11081082 PMC8393342

[6] Edwards MJ , Adams RA, Brown H, Pareés I, Friston KJ. A Bayesian account of “hysteria”. Brain. 2012;135:3495–3512. 22641838 10.1093/brain/aws129 PMC3501967

[7] Larner AJ . Towards a neural network hypothesis for functional cognitive disorders: an extension of the Overfitted Brain Hypothesis. Cogn Neuropsychiatry. 2022 Mar 21:1–8. doi:10.1080/13546805.2022.2054694. 35306961

